# All-Inorganic Lead-Free Perovskite Variant Nanocrystals for Advanced Photonic Applications

**DOI:** 10.3390/s26082470

**Published:** 2026-04-17

**Authors:** Kaixuan Ni, Wei Zhou, Xiaoxiao Zhuang, Xiujuan Zou

**Affiliations:** College of Electronic and Optical Engineering and College of Flexible Electronics (Future Technology), Nanjing University of Posts and Telecommunications, Nanjing 210023, China; b23021724@njupt.edu.cn (K.N.); 1224025339@njupt.edu.cn (X.Z.)

**Keywords:** lead-free perovskite variants, component regulation, tunable optoelectronic properties, self-trapped exciton luminescence, photonic devices

## Abstract

Recently, lead-free metal halide perovskite variant nanocrystals (NCs) have emerged as promising alternatives to their lead-based counterparts, with tunable optoelectronic properties achievable through structural and compositional engineering. Their tunable bandgaps, near-unity quantum yields, solution-processable synthesis routes, and intrinsic environmental benignity render them attractive candidates for a broad range of optoelectronic applications. This review comprehensively summarizes recent advances in perovskite-derived NCs, including diverse synthetic strategies, as well as structural and compositional engineering approaches for optimizing their photophysical properties. Additionally, this review critically discusses the emerging applications of lead-free metal halide perovskite variants, such as solid-state lighting, high-sensitivity photodetection, and advanced radiation imaging. This review aims to provide in-depth insight into the structure–composition–performance relationship of lead-free perovskite variant NCs and pave the way for next-generation eco-friendly optoelectronic materials and devices.

## 1. Introduction

Over the past decade, lead halide perovskites (LHPs) with the general formula APbX_3_ (A = CH_3_NH_3_^+^, CH(NH_2_)^2+^, or Cs^+^; X = Cl, Br, or I) have attracted great research interest owing to their exceptional optoelectronic properties, including strong optical absorption, tunable bandgaps, high carrier mobility and compatibility with solution-based large-area fabrication [1]. Remarkably, perovskite solar cells now exceed 26% power conversion efficiency (PCE), while blue and red perovskite light-emitting diodes (LEDs) reach 26.4% and 32.1% external quantum efficiencies (EQEs), respectively, gradually approaching their physical limits. Parallel to these advancements, extended studies on LHPs have significantly enriched the field of colloidal semiconductor nanocrystals (NCs) [2,3].

Since the first report in 2014, LHP NCs have emerged as star materials due to their near-unity photoluminescence quantum yield (PLQY), narrow-band emission, and facile tunability via halide composition adjustment or dimensional control [4]. Beyond these appealing optical attributes, they exhibit remarkable defect tolerance, an advantage over conventional semiconductor NCs (e.g., CdSe and InP), where structural defects exert a negligible impact on their optical and electronic properties. Leveraging these merits, research has focused on the rational synthesis of diverse LHP NCs, systematic exploration of their photophysical behaviors, and their integration into high-performance optoelectronic devices [5]. However, the following two critical bottlenecks hinder their practical application: (1) poor structural stability against moisture, heat, and light, leading to phase transition or decomposition and (2) lead ion toxicity, which poses substantial environmental and biological risks for consumer electronics.

**Figure 1:**
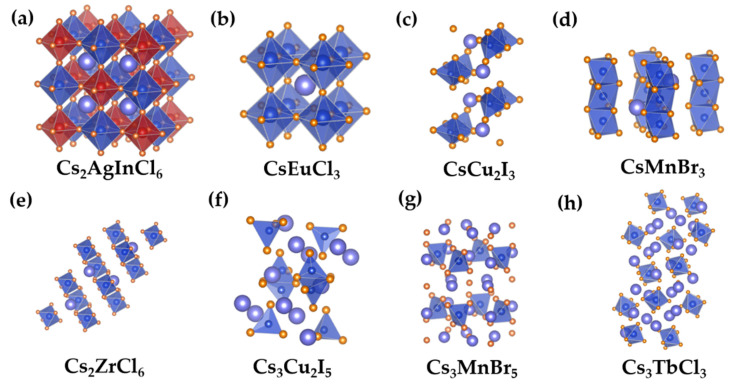
Typical crystal structures of LF-PNCs with 3D, 1D, and 0D configurations. (**a**,**b**) Crystal structures of 3D Cs_2_AgInCl_6_ and CsEuCl_3_; (**c**,**d**) crystal structures of 1D CsCu_2_I_3_ and CsMnBr_3_; (**e**–**h**) crystal structures of 0D Cs_2_ZrCl_6_, Cs_3_Cu_2_I_5_, Cs_3_MnBr_5_ and Cs_3_TbCl_6_, respectively.

To address these limitations, lead-free perovskite variants have emerged as promising next-generation optoelectronic materials, leveraging compositional and structural modulation of the traditional perovskite octahedral framework to enhance stability and eliminate toxicity (Figure 1). This approach aligned with the development trend of low-dimensional metal halides, where structural design and component optimization are core strategies for performance improvement. A-site engineering, for instance, enables versatile tuning via the incorporation of Cs^+^ and Rb^+^ ions in (Cs_x_Rb_1−x_)_3_InCl_6_ NCs, resulting in more regular nanocrystalline morphologies and enhanced luminescence efficiency [6]. As the central strategy for lead replacement, B-site substitution facilitates the rational design and performance regulation of lead-free perovskite variant NCs through the following three well-established pathways: (1) monovalent/trivalent cation co-substitution (e.g., Cs_2_AgBiBr_6_, Cs_2_AgInCl_6_) constructs three-dimensional (3D) cubic perovskite structures, where alternating occupancy of B-sites by two cations maintains charge balance while preserving the intrinsic 3D network topology (Figure 1a) [7]; (2) direct substitution with divalent cations (e.g., Cd^2+^, Eu^2+^, Sn^2+^) in systems such as CsCdCl_3_ and CsEuCl_3_ minimizes lattice distortion due to comparable ionic radii with Pb^2+^, ensuring structural integrity (Figure 1b) [8]; (3) development of tetravalent cation-based vacancy-ordered perovskites (e.g., Cs_2_ZrCl_6_, Cs_2_SnCl_6_) achieves enhanced chemical stability via selective cation site removal from the parent 3D framework (Figure 1c) [9,10]. Notably, these substitution routes enable precise tuning of optical performance through the synergistic effects of cation ionic radius differences and orbital hybridization, laying a solid structural foundation for the application of lead-free perovskite variant NCs in high-performance optoelectronic devices.

Complementing cation engineering, X-site halogen engineering via rational incorporation of mixed halides (Cl^−^, Br^−^, I^−^) enables continuous tuning of the optical bandgap across the ultraviolet-to-near-infrared spectral range [4]. Notably, in synthetic practice, halogen precursors such as trimethylsilyl halides (TMSX, X = Cl, Br, I) provide a versatile route to precise control over halide composition. The halogen engineering strategy not only overcomes the limitations associated with the direct synthesis of target mixed-halide compositions but also substantially expands experimental flexibility. Consequently, it enables the tailored design of lead-free halide perovskite variant NCs with optimized optical and optoelectronic properties for advanced photonic applications [11,12].

It is worth noting that while the periodic table offers a wide spectrum of elements theoretically viable for perovskite matrix construction, experimental investigations have corroborated that only a restricted subset exhibits practical efficacy in forming thermally and chemically robust perovskite lattices. Fortunately, lanthanide (Ln) elements demonstrate remarkable compatibility as B-site cations in octahedral coordination geometries [13,14]. This compatibility originates from their optimal ionic radii closely analogous to that of Pb^2+^ ions, alongside their superior oxidative stability. As a distinctive class of lead-free perovskite variants, the synergy between the 4f electronic configurations of Ln ions and the perovskite lattice confers unique photophysical properties, endowing them with substantial application potential in targeted fields including high-efficiency light-emitting diodes (LEDs), scintillators, and photovoltaics [15].

This review systematically summarizes the latest progress in lead-free perovskite variant NCs, emphasizing the development of novel lead-free systems via component substitution and breakthroughs in performance bottlenecks through luminescence mechanism investigations. The advanced applications of these NCs as optoelectronic materials in solid-state lighting, information encryption, high-energy ray detection, and medical imaging are further explored. By connecting material innovations to practical device performance, this review aims to establish a foundational understanding and offer practical guidelines for this rapidly evolving field, with the goal of guiding future research and application development.

## 2. Compositional and Structural Engineering

Lead-free metal halide perovskite variants have garnered significant attention in the field of optoelectronics. Recent advances in their nanocrystalline forms have shown particularly encouraging progress [16]. In general, due to the absence of degradation pathways associated with organic cations, all-inorganic halide perovskite materials exhibit improved chemical stability compared to their hybrid analogs [17,18,19]. Furthermore, nanocrystalline materials offer compelling advantages such as excellent spectral tunability, ease of chemical modification, straightforward synthesis, and compatibility with miniaturized integration, positioning them as highly promising candidates for next-generation photonic and electronic devices [16].

### 2.1. Indium-Based NCs with Bright Multimode Emission

**Figure 2:**
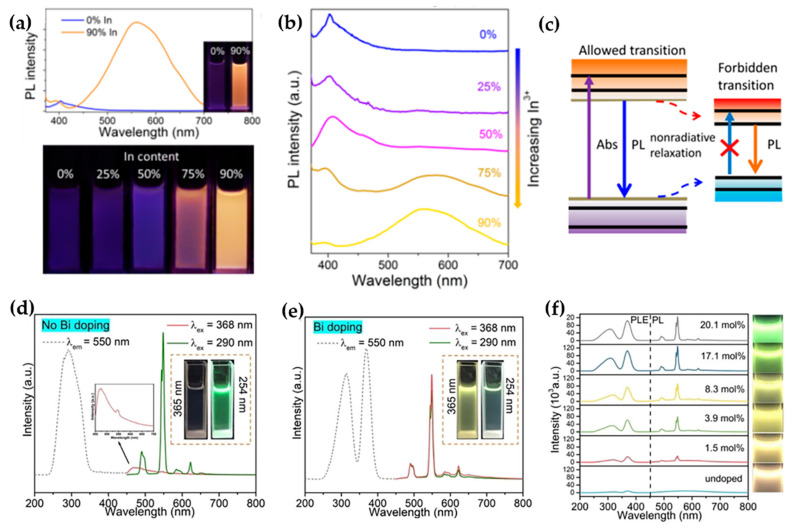
(**a**) PL spectra of Cs_2_AgIn_x_Bi_1−x_Cl_6_ NCs with varying In content [22]; (**b**) PL spectra of Cs_2_AgIn_x_Bi_1−x_Cl_6_ NCs with x = 0.75 and 0.9 [22]; (**c**) direct band gap Cs_2_AgIn_x_Bi_1−x_Cl_6_ (x = 0.75 and 0.9) NCs [22]; (**d**) excitation and emission spectra of the undoped Cs_2_Ag(In_0.829_Tb_0.171_)Cl_6_ sample [21]; (**e**) excitation and emission spectra of the Bi-doped Cs_2_Ag(In_0.829_Tb_0.171_)Cl_6_ sample [21]; (**f**) PLE and PL spectra of Cs_2_Ag(In_1−x_Tb_x_)Cl_6_ NCs with varying Bi doping [21]. The figures were adapted from the cited references.

Compared to their lead-based counterparts, indium-based halide derivatives exhibit superior stability under moisture, light, and thermal stress. Furthermore, these materials typically possess direct bandgap, enabling efficient and tunable emission through simple doping or alloying strategies [20,21]. In 2018, Yang et al. reported the synthesis of lead-free, direct bandgap double-perovskite Cs_2_AgInCl_6_ NCs with bright dual-color emission (Figure 2a) [22]. Utilizing an In/Bi alloying strategy, this work realizes a continuous transition from an indirect to a direct bandgap in lead-free perovskite NCs. The resulting direct-bandgap NCs achieved a high PLQY of up to 36.6% in the violet spectral region, with an absorption cross-section three times greater than that of their indirect-bandgap counterparts. Notably, this system displays unique dual-band emission (violet at ~395 nm and orange at ~570 nm). Na^+^ alloying and Bi^3+^ co-doping were established as another highly effective regulatory approach. By breaking the lattice inversion symmetry and confining the spatial distribution of STEs, this method enhanced the luminescence efficiency of the In-based material by three orders of magnitude to 86%, enabling efficient white-light emission (Figure 2b). These luminescence modulation strategies not only greatly improve optical performance but also unveil significant potential for tailoring emission properties through bandgap engineering and defect control (Figure 2c).

Subsequently, Liu et al. achieved multi-dimensional luminescence modulation and significant performance enhancement by co-doping Tb^3+^/Bi^3+^ into Cs_2_AgInCl_6_ NCs [21]. Leveraging the sensitization effect of Bi^3+^, the effective excitation wavelength for Tb^3+^ emission was successfully redshifted from its intrinsic 290 nm to 368 nm (Figure 2d,e). This shift enables perfect spectral matching with commercial near-ultraviolet LED chips, significantly enhancing the material’s practicality for applications. Concurrently, an efficient energy transfer pathway was established from the host’s STEs to the Tb^3+^ ions, substantially boosting the overall energy transfer efficiency. Furthermore, by precisely adjusting the Tb^3+^ doping concentration, continuous tuning of the emission color from green to orange was achieved, demonstrating excellent luminescence tunability (Figure 2f).

In addition to 3D, 0D In-based perovskite derivatives have also attracted considerable attention due to their unique localized electronic structures. In 2021, our group reported a series of Sb^3+^ doped all-inorganic lead-free 0D In-based perovskite variant NCs (Rb_x_Cs_1−x_)_3_InCl_6_, synthesized via a modified hot-injection method [6]. In the anhydrous form A_3_InX_6_ (A = Rb or Cs), the [InX_6_]^3−^ octahedra exhibit high symmetry (space group C2/c), where each In^3+^ ion coordinates with six X^−^ ions to form a regular octahedral configuration. In this work, multidimensional pathways for the performance regulation of 0D perovskite variants were systematically explored. Firstly, via an A-site cation alloying strategy, the Rb/Cs ratio (0 ≤ x ≤ 1) in (Rb_x_Cs_1−x_)_3_InCl_6_ was tuned to effectively passivate bulk defects and significantly enhance the air stability of the NCs. Moreover, the incorporation of Sb^3+^ ions introduced a [SbCl_6_]^3−^ octahedral sublattice, which not only created a new absorption band but also led to dopant-induced self-trapped excitons, resulting in bright green emission with microsecond-scale lifetime. Furthermore, a two-step method involving variable-temperature hot injection followed by saturated vapor post-treatment was also employed to synthesize water-coordinated In-based Cs_2_InCl_5_(H_2_O):Sb NCs for the first time. The coordinated water replaces Cl^−^ to form the water-coordinated octahedra, which not only optimizes the lattice environment and effectively passivates surface defects, but also significantly enhances exciton–phonon coupling, achieving a PLQY of 95.5%. By replacing Cl^−^ with Br^−^, Cs_2_InBr_5_(H_2_O):Sb NCs were also successfully prepared, and the emission color continuously redshifted from yellow (Cl-based, 550 nm) to orange. This investigation demonstrates that the electronegativity difference among halide ions can be used to modulate the bandgap, enabling emission tuning across the visible range and offering potential for full-color display applications.

### 2.2. Switchable Emissive Manganese-Based Metal Halide

Manganese-based metal halide NCs have emerged as a promising class of all-inorganic lead-free perovskite variants, addressing the critical limitations of lead toxicity and poor color tunability in lead-free perovskites. Their unique capability of switchable emission, rooted in the intrinsic sensitivity of Mn^2+^ luminescence to crystal field effects and coordination environments, has opened new avenues for advanced photonic applications ranging from high-resolution displays to anti-counterfeiting and smart light-emitting diodes (LEDs).

**Figure 3:**
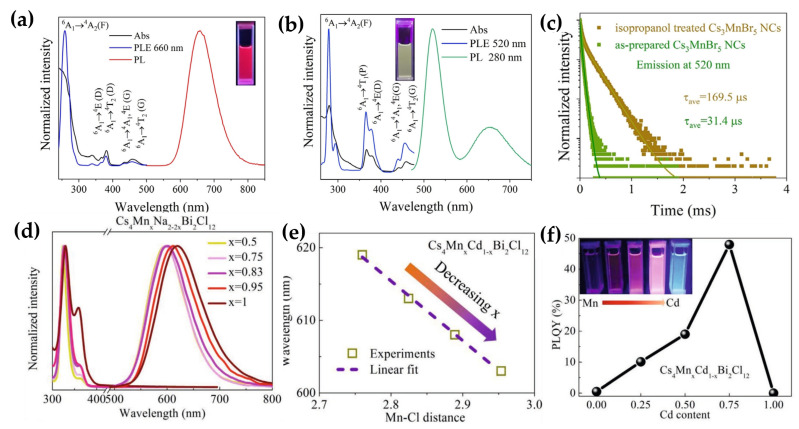
(**a**) UV–Vis absorption, PLE, and PL spectra of CsMnBr_3_ NCs [23]; (**b**) UV–Vis absorption, PLE, and PL spectra of as-prepared Cs_3_MnBr_5_ NCs [23]; (**c**) TR-PL decay curves of isopropanol-treated and as-prepared Cs_3_MnBr_5_ NCs [23]; (**d**) normalized absorption and PL spectra of Cs_4_Mn_x_Na_2−2x_Bi_2_Cl_12_ NCs [25]; (**e**) correlation between Mn–Cl bond distances and PL-peak position for Cs_4_Mn_x_Cd_1−1x_Bi_2_Cl_12_NCs [25]; (**f**) PLQY of Cs_4_Mn_x_Cd_1−x_Bi_2_Cl_12_NCs [25]. The figures were adapted from the cited references.

Recently, Kong et al. achieved tunable red/green/blue emission with high color purity via an isopropanol-triggered phase transition strategy in Mn-based metal halide NCs [23]. By controlling the Cs/Mn feeding ratio via a hot-injection method, they successfully synthesized octahedrally coordinated 1D CsMnBr_3_ NCs exhibiting red emission and tetrahedrally coordinated 0D Cs_3_MnBr_5_ NCs emitting green light (Figure 3a–c). The phase transition, triggered by isopropanol treatment, induced the dissolution of MnBr_2_ from 1D CsMnBr_3_, transforming it into the more stable 0D Cs_3_MnBr_5_ structure. This process resulted in a red-to-green emission shift alongside improved crystallinity and enhanced photoluminescence efficiency. Notably, in a humid environment, the introduction of water molecules further modulates the coordination environment of Mn^2+^ ions, converting both CsMnBr_3_ and Cs_3_MnBr_5_ into 0D Cs_2_MnBr_4_·2H_2_O NCs. This final transformation enables efficient blue emission centered at 440 nm. The switchable luminescence of the series of Mn-based metal halide NCs originates from the parity-forbidden d-d transition of Mn^2+^ ions (^4^T_1_→^6^A_1_). The emission energy and spectral profile are predominantly governed by the crystal field strength and the specific coordination geometry of the Mn^2+^ centers. As a fundamental design principle, the Mn^2+^ coordination environment can be precisely tailored between octahedral ([MnBr_6_]^4−^) and tetrahedral ([MnBr_4_]^2−^) configurations, yielding distinct emission characteristics. This work establishes a clear “structure/property” relationship between the structural dimensionality/coordination environment and the resultant emission color.

Similarly, Mohammed et al. confirmed that the luminescence of 1D chain-structured CsMnBr_3_ NCs originates from the low-lying excited state (^4^T_1_) of Mn^2+^ through femtosecond transient absorption and time-resolved photoluminescence spectroscopy, with an ultrafast lifetime of ∼605 ps [24]. The exceptionally short lifetime is attributed to the strong interionic coupling induced by the extremely short Mn-Mn distance (∼2.7 Å) within the crystal structure. These findings provide a foundation for developing novel lead-free luminescent materials that combine high efficiency with ultrafast optical response.

Earlier research largely concentrated on low-dimensional Mn-based perovskite derivatives. Recently, Bai et al. reported the first synthesis of 3D colloidal vacancy-ordered quadruple perovskite Cs_4_Mn(Sb_x_/Bi_1−x_)_2_Cl_12_ NCs, demonstrating the feasibility of constructing complex ordered vacancy structures at the nanoscale and significantly expanding the materials library of lead-free perovskites [25]. This structure is derived from the A_2_M′M″X_6_ by substituting two M’^3+^ sites with one divalent cation (M^2+^) and one vacancy (Figure 3d). Through alloying with trivalent (Sb^3+^/Bi^3+^), divalent (Cd^2+^/Mn^2+^), and monovalent (Na^+^) metals, the Mn-Cl bond lengths around Mn^2+^ were linearly tuned, thereby modifying the crystal field strength and enabling continuous adjustment of the Mn^2+^ d-d emission peak across the range of 590–640 nm (Figure 3e). Notably, Cd^2+^ alloying resulted in a 96-fold enhancement in photoluminescence quantum yield and a 77-fold increase in lifetime (Figure 3f). This improvement was attributed to a precise “trap engineering”, with temperature-dependent photoluminescence and femtosecond transient absorption spectroscopy clearly revealed that alloying eliminates an ultrafast (1.4 ps) carrier trapping channel while simultaneously enhancing crystallinity, thereby collectively suppressing non-radiative recombination.

### 2.3. Copper(I)-Based NCs for near Unity PLQY

Copper(I)-based halide perovskites have garnered considerable attention in NC research owing to their distinctive properties, including low-dimensional electronic structures, high exciton binding energy, broadband emission, and environmental friendliness. Particularly, their multi-mode emission and near-unity PLQY make them highly promising for applications in light-emitting diodes, scintillation imaging, and fluorescence anti-counterfeiting [26].

**Figure 4:**
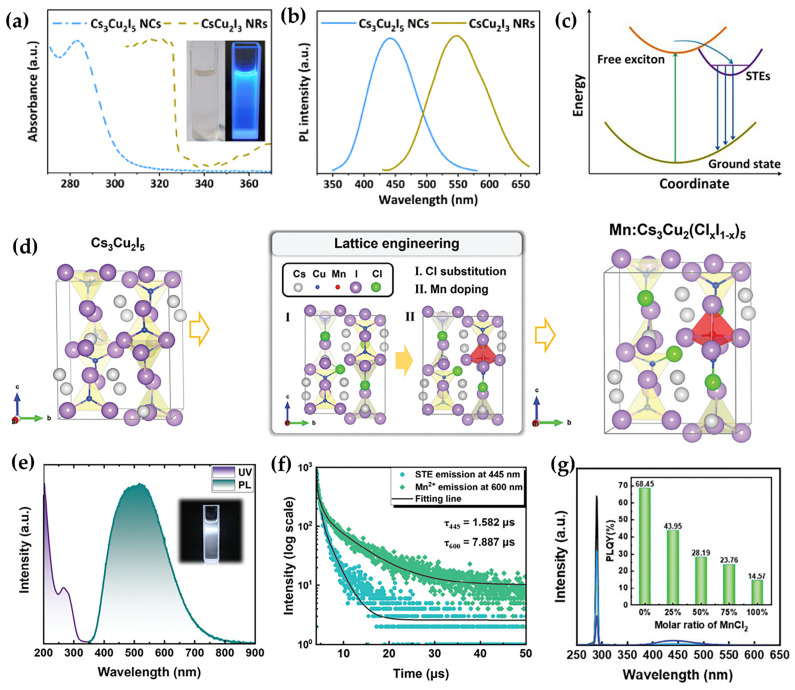
(**a**) UV-Vis absorption spectra of CsCu_2_I_3_ NRs and Cs_3_Cu_2_I_5_ NCs [27]; (**b**) PL spectra of CsCu_2_I_3_ NRs and Cs_3_Cu_2_I_5_ NCs [27]; (**c**) schematic illustration of the photophysical processes in Cs_3_Cu_2_I_5_ NCs [27]; (**d**) mechanistic diagram demonstrating the relationship between Cl substitution and Mn-doping based on a lattice engineering strategy [28]; (**e**) absorption and PL spectra of Mn-doped NCs with a MnCl_2_ molar ratio of 50% [28]; (**f**) TRPL decay dynamics spectra monitored at 445 nm and 605 nm [28]; (**g**) PLQY spectra and corresponding values of NCs with varying MnCl_2_ molar ratios [28]. The figures were adapted from the cited references.

Hot-injection method, which enables rapid nucleation and controlled crystal growth, represents a primary strategy for synthesizing Cu-based NCs. In 2019, Cheng et al. successfully synthesized 1D CsCu_2_I_3_ nanorods and 0D Cs_3_Cu_2_I_5_ NCs for the first time via a modified hot injection method (Figure 4a) [27]. The crystal structure of 0D Cs_3_Cu_2_I_5_ is composed of isolated [Cu_2_I_5_]^3−^ dimeric units, which contain Cu^+^ sites in two distinct coordination geometries of tetrahedral and trigonal. These structural motifs are fully separated in space by Cs^+^ cations, resulting in a characteristic localized electronic structure. The corresponding 0D NCs exhibit intense blue emission with a PLQY of 67%. In contrast, the 1D nanorods feature a double-chain structure extending along the c-axis, formed by edge-sharing [CuI_4_]^3−^ tetrahedra. This structural arrangement leads to exciton delocalization along the inorganic chains, which significantly reduces the electron-phonon coupling. Consequently, non-radiative recombination dominates the relaxation pathway, yielding only weak yellow emission at 553 nm with a quantum efficiency of merely 5% (Figure 4b). This pronounced difference in photophysical behavior, spanning from the 0D localized states to the 1D delocalized states, clearly demonstrates how structural dimensionality governs the formation energy of STEs and modulates the channels for radiative recombination (Figure 4c). Subsequently, Li et al. employed a room-temperature antisolvent method to synthesize the full series of Cs_3_Cu_2_X_5_ NCs by systematically varying the halide composition. This approach significantly streamlined the synthesis process and minimized energy consumption and specialized equipment requirements compared to conventional synthesis techniques [27].

Nevertheless, pure-phase NCs generally suffer from intrinsic drawbacks such as low PLQY and narrow spectral tunability, primarily due to their lower crystallinity and increased lattice defects compared to single-crystal counterparts. These limitations have hindered their practical application. Fortunately, the combination of ion doping and lattice engineering has emerged as a key strategy to address these challenges. By carefully selecting dopant ions and precisely modulating the lattice structure, the optical performance, structural stability, and application compatibility of materials can be comprehensively enhanced. For instance, in 2025, Chen et al. demonstrated selective substitution of Cs^+^ sites with Rb^+^ ions in Cs_3_Cu_2_I_5_ nanocrystals [29]. As Rb^+^ and Cs^+^ belong to the same group (IA) and share identical valence states with similar chemical properties, the doping process did not compromise lattice stability. Additionally, the smaller ionic radius of Rb^+^ (147 pm) induced a lattice contraction. This contraction not only enhanced the exciton-photon coupling but also significantly suppressed non-radiative recombination pathways. At an optimal Rb doping level of 5%, the PLQY increased from 85% for the pure phase to 96%. Moreover, the material exhibited substantially improved air stability, retaining 97.18% of its initial photoluminescence intensity after six days.

Unlike the straightforward isovalent substitution in Rb doping, heterovalent ion doping in NCs faces the fundamental challenge of lattice compatibility. For instance, the ionic radius of Mn^2+^ (83 pm) is larger than that of Cu^+^ (77 pm), making it difficult to directly incorporate Mn^2+^ into the tetrahedral [CuX_4_] and trigonal planar [CuX_3_] sites of Cs_3_Cu_2_I_5_. To address this, Li et al. introduced a lattice-engineering strategy by partially substituting I^−^ with Cl^−^ in Cs_3_Cu_2_I_5_ to form Cs_3_Cu_2_(Cl_x_I_1−x_)_5_. The smaller ionic radius of Cl^−^ (181 pm) compared to I^−^ (220 pm) induces moderate lattice contraction, thereby creating effective doping sites and enabling uniform incorporation of Mn^2+^ (Figure 4d) [28]. This optimization not only overcomes the doping barrier but also establishes an efficient energy-transfer pathway. The pristine Cs_3_Cu_2_I_5_ exhibits blue STE emission (~450 nm), while the introduction of Mn^2+^ adds a yellow-orange emission (~600 nm) originating from Mn^2+^ d-d transitions. The complementary emissions enable the generation of full-spectrum white light, with the photoluminescence spectrum covering the entire visible range and exhibiting a FWHM of 212.83 nm (Figure 4e). By precisely controlling dopant selection and lattice engineering, the functional properties of copper-based halide NCs can be selectively enhanced (Figure 4f,g). This capability paves the way for their versatile applications in healthcare, lighting, and security, and sets a clear direction for the future development of eco-friendly, lead-free halide materials.

In lead- and tin-based perovskite NCs (e.g., APbX_3_ or ASnX_3_), the narrow emission linewidth (small FWHM) predominantly originates from band-edge recombination of free excitons within a highly symmetric lattice with delocalized electronic states [30,31]. By contrast, lead-free perovskite variants such as Cs_3_Cu_2_X_5_ exhibit intrinsically broad emission (large FWHM), which is fundamentally linked to their low-dimensional crystal structure comprising isolated [Cu_2_X_5_]^3−^ units [32]. This structural motif enables strong electron-phonon coupling and significant lattice relaxation upon photoexcitation, favoring the formation of STEs. The radiative recombination of STEs, involving substantial excited-state structural distortion, gives rise to broadband emission with large Stokes shifts. Importantly, the emission broadening cannot be ascribed solely to STE formation. The pronounced electronic localization inherent to the 0D (or quasi-0D) framework further amplifies sensitivity to local structural fluctuations, thereby contributing to additional spectral broadening.

### 2.4. Rare-Earth-Based Metal Halide NCs as a New Hot Topic

Emerging as a promising candidate for advanced optoelectronics, all-inorganic rare-earth based metal halide NCs are distinguished by their unique luminescent properties, precisely tunable optics, and robust stability. These materials present a compelling, non-toxic alternative to lead-based perovskites by leveraging the characteristic 4f-4f/5d-4f transitions of lanthanide ions to achieve narrow-band emission across a broad spectrum from UV to NIR. These attributes lay a solid foundation for their deployment in next-generation technologies, including high-color-purity displays, biological sensing, secure information encryption, and high-resolution radiographic imaging [15].

**Figure 5:**
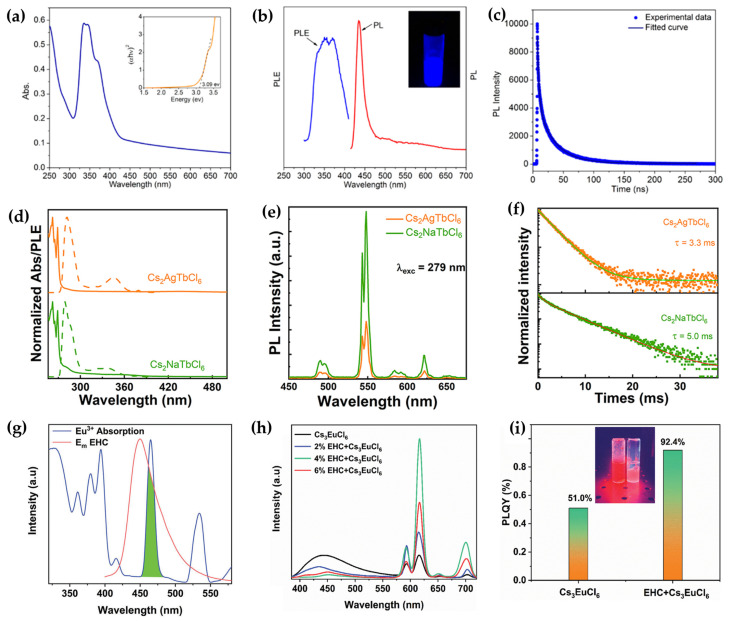
(**a**) Optical absorption of CsEuCl_3_ NCs [33]; (**b**) PL (red) and PLE (blue) spectra collected at 435 nm [33]; (**c**) TRPL at 435 nm [33]. Copyright 2020 Nano Lett; (**d**) absorption (solid lines) and PLE (dashed lines) spectra of Cs_2_BTbCl_6_ (B = Ag or Na) NCs [34]; (**e**) PL spectra of Cs_2_BTbCl_6_ (B = Ag or Na) NCs [34]; (**f**) TRPL of Cs_2_BTbCl_6_ (B = Ag or Na) NC [34]; (**g**) emission spectra of Eu^3+^ in Cs_3_EuCl_6_ NCs (red line) and corresponding absorption spectra (blue line) of EHC [35]; (**h**) emission spectra of pure Cs_3_EuCl_6_ NCs and Cs_3_EuCl_6_ NCs treated with different amounts of EHC [35]; (**i**) PLQY of Cs_3_EuCl_6_ NCs and EHC-treated Cs_3_EuCl_6_ NCs [35]. The figures were adapted from the cited references.

Rare-earth metal halide NCs exhibit unique multimodal emission, originats from distinct luminescence mechanisms including band-edge emission, f-f/d-f transitions of rare earth ions, and STEs. Yang et al. reported CsEuCl_3_ NCs synthesized via the hot-injection method, in which EuCl_3_ served as the precursor and oleylamine acted as a reducing agent, effectively reducing Eu^3+^ to Eu^2+^ during the reaction [33]. This approach overcomes the challenges of poor solubility and oxidation susceptibility commonly associated with rare-earth precursors. Efficient blue emission is achieved through the 5d→4f transition of Eu^2+^, with an FWHM of only 19 nm, one of the narrowest reported among lead-free perovskite NCs to date (Figure 5a–c). Such high color purity is crucial for enabling high-resolution, high-saturation display applications. Furthermore, surface treatment with an ionic ligand (1-butyl-1-methylpyrrolidinium chloride) resulted in approximately threefold enhancement in PLQY. This improvement suggests that the ligand reduces non-radiative recombination by supplementing chloride ions and passivating surface halogen vacancies, offering a new direction for the surface engineering of rare-earth perovskite NCs.

Subsequently, Wu and co-workers reported phase-pure CsEuBr_3_ NCs obtained through halide substitution from Cl to Br [36]. This compositional modulation effectively mitigates the deep-level defects associated with Cl, thereby enhancing emission efficiency. Under ultraviolet excitation, CsEuBr_3_ NCs show intense blue emission centered at 421 nm, together with a narrow FWHM of 21 nm and a PLQY of 70%, reflecting their high color purity and efficient emissive characteristics. The exciton binding energy was determined to be 43 meV, which is comparable to that of lead-based NCs and points to good exciton stability. However, despite these attractive optical features, Eu^2+^-based NCs still face a major challenge in terms of stability, owing to their proneness to oxidation and marked hygroscopicity.

In parallel to Eu^2+^-based systems, Song and co-workers developed Cs_2_NaEuCl_6_ NCs by incorporating trivalent europium as a lattice-forming cation, and further demonstrated its efficient application in light-emitting diodes [37]. Notably, unlike Eu^2+^-based NCs, the broadband emission of Cs_2_NaEuCl_6_ originates from STEs rather than band-edge recombination or defect-related emission. This STE-dominated emission mechanism is supported by temperature-dependent PL measurements, which reveal a gradual blueshift of the broadband emission, an increase in the FWHM, and continuous thermal quenching of the emission intensity with increasing temperature. Femtosecond transient absorption spectroscopy further identifies a photoinduced absorption band in the 340–540 nm range, providing direct evidence for STE formation in the lattice. Furthermore, the large Huang-Rhys factor (S = 10.88) indicates pronounced electron-phonon coupling, which plays a critical role in exciton self-trapping. More importantly, the Cs_2_NaEuCl_6_ NCs exhibit markedly improved stability compared with Eu^2+^-based counterparts, retaining 83% of their initial PL intensity after 500 h of continuous ultraviolet irradiation.

In 2023, Zhou et al. reported the synthesis of rare-earth-based Cs_2_AgTbCl_6_ and Cs_2_NaTbCl_6_ NCs [34]. Both Tb-based NCs exhibit uniform sizes, high crystallinity, and bright narrow-band green emission. Notably, Cs_2_NaTbCl_6_ displays superior luminescence performance compared to Cs_2_AgTbCl_6_, with PLQY three times higher (Figure 5d,e). This enhancement originates from the highly localized charge distribution within the [TbCl_6_]^3−^ octahedra in Cs_2_NaTbCl_6_, which facilitates Cl^−^→Tb^3+^ charge transfer and promotes f–f emission of Tb^3+^ (Figure 5f). In contrast, the covalent character of the Ag^+^-Cl^−^ bond in Cs_2_AgTbCl_6_ leads to charge delocalization toward Ag^+^, weakening the coupling between Cl^−^ and Tb^3+^ and thus impairing energy transfer to the Tb^3+^ centers. This work provided the first electronic-structure-level insight into how the nature of B-site cations critically governs the energy transfer efficiency to rare-earth luminescent centers. Subsequently, we further investigated the influence of crystal structure on the properties of rare-earth halide NCs and reported the synthesis of 0D Cs_3_TbCl_6_ NCs via the hot-injection method [38]. Owing to the more strongly localized excitons and greater lattice tolerance inherent to the 0D structure, Cs_3_TbCl_6_ achieves PLQY twice that of the 3D Cs_2_NaTbCl_6_. Moreover, in Sb^3+^-doped 0D Cs_3_TbCl_6_, a thermally promoted energy transfer pathway was constructed via STE states induced by [SbCl_6_]^3−^ units, which significantly enhanced the luminescence efficiency of Tb^3+^, yielding a quantum yield of 48.1%.

Beyond doping with ns^2^-type ions, Sun et al. further optimized the performance of 0D rare-earth halides through surface modification with coumarin-based small molecules [39]. A series of 0D Cs_3_LnCl_6_ NCs modified with ethylhexyl substituted coumarin (EHC) was synthesized, achieving broadly tunable emission from blue to near-infrared across the entire spectrum. The EHC molecule coordinates to unsaturated lanthanide ions via its C=O group, reducing surface defects and thereby enhancing both the PLQY and crystallinity (Figure 5g,h). Simultaneously, its energy levels align well with those of the lanthanide ions, acting as an efficient energy-transfer bridge and raising the energy-transfer efficiency to 93.2% (Figure 5i). Moreover, the hydrophobic nature of EHC improves the moisture stability of the material, while post-treatment with EHC enhances thin-film quality, leading to improved device performance. Through the synergistic mechanisms of defect passivation, energy bridging, and interface regulation, the coumarin small molecule EHC comprehensively enhances the optical properties, environmental stability, and electroluminescent device potential of lanthanide-based lead-free perovskite NCs. This work provides an important material design and processing strategy for developing efficient, stable, and full-color display technologies.

The structural diversity and doping flexibility of rare-earth-based metal halides render them highly advantageous for achieving multicolor tunable emission and near-infrared-excited upconversion luminescence. Han et al. reported the upconversion (UC) luminescence based on a novel 0D metal halide Cs_3_GdCl_6_, utilizing Yb^3+^ as a sensitizer and Er^3+^, Ho^3+^, and Tm^3+^ as activators to realize distinct UC emission colors [40]. In the Yb^3+^/Er^3+^ co-doped system, Yb^3+^ absorbs 980 nm photons and transfers energy to Er^3+^. Subsequent energy transfer UC or excited-state absorption promotes electrons to the ^4^F_7/2_ level, followed by radiative transitions that generate green emission; the red emission originates from the ^4^F_9/2_ level via multiphonon relaxation. Furthermore, Cs_3_GdCl_6_:Yb^3+^,Er^3+^ exhibits unique hydrochromic luminescence properties, wherein the UC emission color gradually changes from green to red upon exposure to moisture.

In 2025, Kong et al. systematically investigated the UC luminescence properties and their synergistic role in multimodal emission in Cs_2_NaYbCl_6_:Er^3+^ NCs [41]. In this system, Yb^3+^ serves simultaneously as a host cation and a sensitizer, while Er^3+^ acts as the activator. The most prominent innovation of this material lies in the successful integration of five distinct luminescence modes including upconversion, downshifting, persistent luminescence, thermochromic emission, and hydrochromic emission. Under 980 nm excitation, the UC emission is dominated by the characteristic green luminescence of Er^3+^. Under ultraviolet excitation, efficient blue emission is generated via self-trapped excitons. Upon cessation of X-ray irradiation, the material releases stored charge carriers through shallow trap levels such as chlorine vacancies, resulting in color-tunable persistent luminescence. Moreover, upon immersion in water, the UC channel is reversibly quenched while the blue downshifting emission is retained; the original emission properties are restored after drying, revealing a distinctive hydrochromic response. These luminescence modes can be excited independently using different stimulation sources or switched synergistically under specific conditions, all originating from the same single-phase material. This fully demonstrates the design advantages arising from the combination of the energy level structure of rare-earth ions with host defect engineering, providing an ideal optical platform for constructing anti-counterfeiting and information encryption systems with high encoding capacity and enhanced security.

### 2.5. Synthesis of Lead-Free Perovskite Variant NCs

**Figure 6:**
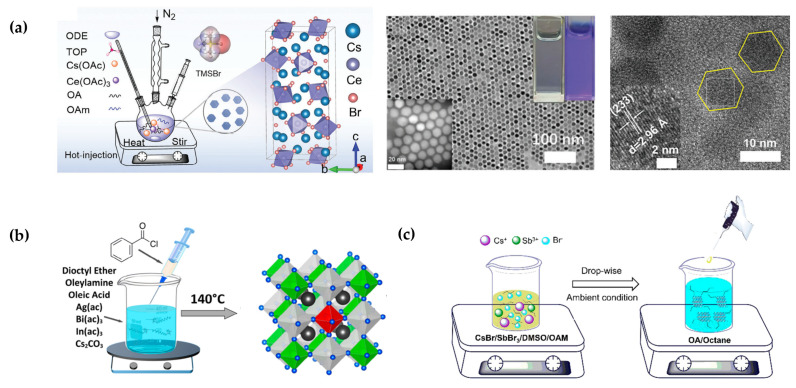
Synthesis methods. (**a**) High-temperature thermal injection synthesis (under vacuum and in a nitrogen atmosphere) [43]. (**b**) Open-air thermal injection synthesis [44]. (**c**) Ligand-assisted reprecipitation method (LAPR) [47].

The synthesis of all-inorganic lead-free perovskite NCs faces challenges distinctly different from those of conventional lead-based systems, primarily arising from the intricate coordination chemistry of lead-free metal ions (e.g., In^3+^, Cu^+^, and Ln^3+^ ions) and their low solubility in precursors [16,42]. These materials require precise control over precursor reactivity and ligand coordination capability to prevent phase separation or morphological degradation. Through synthetic approaches such as hot injection, ligand-assisted reprecipitation (LARP), and heating-up methods, combined with optimization of reaction temperature, precursor ratios, and surface passivation agents, all-inorganic lead-free perovskite NCs with high crystallinity and pure phase have been successfully realized. These materials not only circumvent the toxicity concerns but also exhibit exceptional stability and tunable optoelectronic properties, establishing a solid foundation for their practical application in optoelectronic devices. The hot injection method has become the dominant approach for synthesizing lead-free halide NCs, owing to its atomic-level precision in product regulation and short reaction time. This method involves rapidly injecting one precursor into a hot solution containing metal halides and ligands under an inert atmosphere, where instantaneous supersaturation triggers rapid nucleation and controlled growth (Figure 6a,b) [43,44]. This strategy enables precise morphological control, yielding structures such as quantum dots, nanorods, nanoplatelets, and core–shell heterostructures [45,46]. Moreover, it demonstrates exceptional versatility, being applicable to nearly all lead-free halide systems, including oxidation-sensitive tin-based systems, nucleation-sensitive double perovskites, as well as bismuth/antimony-based, copper-based, and rare-earth-based systems. However, its scale-up potential remains constrained by demanding reaction conditions, reliance on high-boiling-point organic solvents, and complex post-processing procedures.

The LARP method has also been widely adopted for the synthesis of lead-free halide perovskite NCs in recent years. This approach involves rapidly injecting a precursor solution (good solvent such as DMF or DMSO) into a poor solvent containing ligands (e.g., toluene or chloroform) at room or low temperature, where the abrupt change in solvent polarity induces rapid precipitation and NC formation (Figure 6c) [47]. As the reaction proceeds under ambient temperature and pressure and exhibits excellent compatibility with air-stable systems such as bismuth/antimony-based, 0D copper-based, and 3D double perovskites, this method holds substantial promise for large-scale production.

In addition, the heating-up method has also been employed for synthesizing lead-free halide perovskite NCs in highly stable systems. In this approach, metal halide precursors are premixed with ligand solvents such as oleic acid, oleylamine, and 1-octadecene, degassed under vacuum, and then directly heated to the reaction temperature to achieve homogeneous nucleation and growth of NCs [48,49]. Compared to the hot injection method, the heating-up method eliminates the need for a rapid injection step, offering simplified operation and enhanced scalability. However, in lead-free systems, this method is more susceptible to broad size distributions and phase impurities, necessitating precise optimization of reaction parameters to effectively regulate crystallization kinetics and surface chemistry.

To achieve performance breakthroughs and enable scalable fabrication of lead-free halide perovskite NCs, researchers are also actively exploring novel synthetic routes, including interface-confined synthesis, femtosecond laser- or light-induced in situ synthesis, ultrasound-assisted synthesis, and mechanochemical synthesis [50,51,52,53]. These emerging strategies offer viable pathways toward the low-cost, green manufacturing of optoelectronic devices based on lead-free perovskites. Table 1 summarized the photophysical properties of typical lead-free perovskite variant NCs.

## 3. Applications

### 3.1. Solid-State Lighting

In the 21st century, lighting and display technologies are undergoing rapid advancement toward high brightness, wide color gamut, low power consumption, and environmentally benign characteristics. Lead-free perovskite NCs, with their tunable emission, high PLQY, and cost-effective solution-processable fabrication, have successfully addressed the long-standing trade-off between performance and environmental sustainability inherent in conventional semiconductor materials. Capable of functioning as both photoluminescent phosphors for LED down-conversion and as emissive layers in electroluminescent (EL) devices, these nanomaterials are emerging as highly promising candidates for driving the next wave of innovation in lighting and display technologies [7,70].

**Figure 7:**
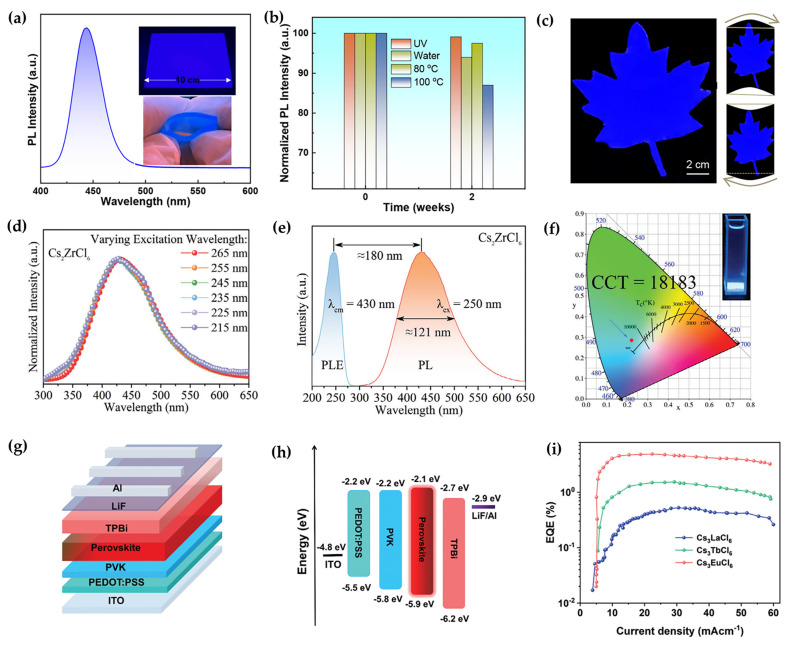
(**a**) Emission spectra of CsEuBr_3_ NCs embedded in a PDMS composite film [71]; (**b**) environmental stability tests of the composite films [71]; (**c**) photographs of a “maple-leaf” patterned film at different folding angles [71]; (**d**) PL spectra of Cs_2_ZrCl_6_ sample with varying excitation wavelength [72]; (**e**) PL and PLE spectra of the Cs_2_ZrCl_6_ [72]; (**f**) color coordinate of the LED device under a current of 15 mA [72]; (**g**) device structure of the PeLEDs [35]; (**h**) schematic energy-level diagram of the PeLEDs [35]; (**i**) EQE versus voltage curves for Cs_3_LaCl_6_, Cs_3_EuCl_6_, and Cs_3_TbCl_6_ based PeLEDs [35]. The figures were adapted from the cited references.

Phosphor-converted LEDs are typically fabricated by integrating commercial ultraviolet or blue chips with metal halide NC phosphors. Due to their tunable bandgaps and near-unity quantum yields, metal halide NC-based LEDs can simultaneously achieve high brightness and precise control over emission wavelength. Among them, deep-blue narrow-band emitters are crucial for enabling wide-color-gamut backlighting in liquid crystal displays; however, the lead-free candidates have long been hindered by low PLQYs and broad emission. In 2024, Li et al. reported a novel deep-blue narrow-band emitter based on lead-free perovskite CsEuBr_3_ NCs (Figure 7a,b) [71]. Using a modified hot-injection method under optimized conditions, CsEuBr_3_ achieved a remarkable PLQY of up to 93.51% and a FWHM of 28.5 nm. This performance sets a new efficiency record for deep-blue lead-free perovskite NCs. Furthermore, the study systematically elucidated the luminescence mechanism through both theoretical and experimental approaches, attributing the emission to Eu-5d → Eu-4f/Br-4p electron transitions. Flexible blue-emitting films were fabricated by embedding CsEuBr_3_ NCs into a polydimethylsiloxane (PDMS) matrix (Figure 7c). These films were successfully integrated into white LEDs, achieving a broad color gamut covering 127.1% of the NTSC standard and 94.9% of Rec. 2020, along with an extended operational half-lifetime of 1677 h. This work not only provides a new material platform for efficient, stable, and environmentally friendly blue-emitting perovskites, but also lays a solid foundation for their practical application in wide-color-gamut display backlighting.

Additionally, vacancy-ordered Cs_2_ZrCl_6_ NCs exhibit bright deep-blue emission originating from STEs. Wei et al. innovatively proposed an InCl_3_-assisted surface defect repair strategy, which effectively suppresses the non-radiative recombination of STEs and endows the material with a high exciton binding energy of 354.68 meV and excellent thermal stability (Figure 7d,e) [72]. With an optimal InCl_3_ addition of 2%, the photoluminescence intensity of the NCs is enhanced by approximately 30%. Furthermore, by embedding the optimized nanocrystals into PDMS, the researchers successfully fabricated a flexible fluorescent film that demonstrates outstanding water resistance and bending durability. This film was subsequently employed to construct a stable blue-emitting LED device (Figure 7f).

White-light emitters hold substantial promise for both lighting and display applications. Achieving bright, broadband white-light emission from environmentally friendly and stable lead-free materials is therefore of significant practical importance. Li et al. reported the room-temperature antisolvent synthesis of a series of Cs_3_Cu_2_X_5_ NCs (X = Cl, Br, I, and mixed halides) [73]. These NCs exhibit a green emission peak at 521 nm with a broad FWHM of 104 nm, characteristic of STE luminescence. Furthermore, by tuning the halide composition, the emission wavelength of this series can be continuously adjusted from 443 to 530 nm, covering the blue to green spectral regions. Concurrently, CsCu_2_I_3_ NCs provide yellow/red emission centered at 575 nm, laying a foundation for full-spectrum light-emitting material systems. By appropriately blending these copper-based NCs, high-quality white light with CIE coordinates of (0.2880, 0.3277) can be achieved.

In addition to their role as photoluminescent materials for down-conversion LEDs, lead-free perovskite NCs have also shown significant progress in the field of electroluminescence in recent years. Wang et al. fabricated a deep-blue light-emitting diode using 0D Cs_3_Cu_2_I_5_ as the emissive layer [74]. Leveraging the STE emission and the enhanced exciton localization inherent to the 0D structure of Cs_3_Cu_2_I_5_, the device achieved efficient deep-blue emission at 445 nm, with an EQE of 1.12% and a half-lifetime of 108 h. The lead-free nature, combined with its outstanding stability, overcomes the long-standing challenges of toxicity and phase instability associated with conventional lead-based blue-emitting perovskites. This work marks the first demonstration of the practical potential of copper-based lead-free perovskites for blue-emitting LEDs. In 2024, Sun et al. successfully fabricated red, green, and blue LEDs based on EHC-modified Cs_3_LnCl_6_ NCs (Figure 7g) [35]. The introduction of EHC not only passivates halide vacancies and lattice defects to suppress non-radiative recombination but also establishes an efficient “host → EHC → rare-earth ion” energy-transfer pathway (Figure 7h), thereby significantly enhancing the overall efficiency and improving electroluminescent performance. Notably, the red LED based on EHC-modified Cs_3_EuCl_6_ exhibits a narrow-band emission with a high color purity (FWHM ≈ 18 nm), achieving an EQE of 5.17% and a maximum luminance of 2373 cd·m^−2^ (Figure 7i).

### 3.2. Optical Detection and Sensing

All-inorganic lead-free perovskite variant NCs have emerged as promising candidates for optical detection and sensing, benefiting from their excellent optical properties, structural stability, and solution processability. To date, they have been successfully applied in multiple sensing scenarios, including photodetection, gas sensing, temperature sensing, and ion detection, with performance comparable or even superior to traditional materials.

**Figure 8:**
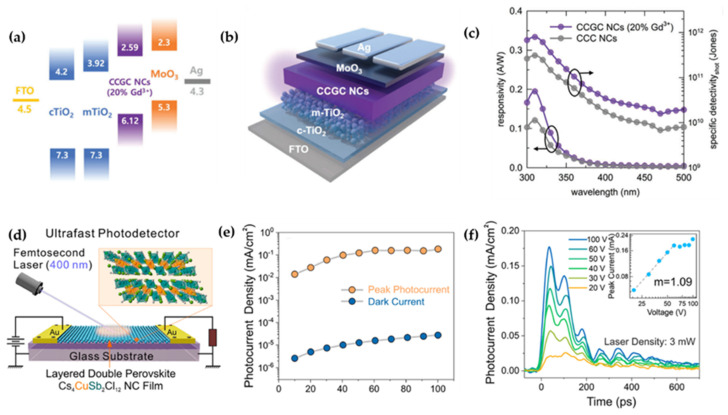
(**a**) Energy level diagram of the PD device [75]; (**b**) schematic illustration of the PD device structure [75]; (**c**) spectral responsivity and specific detectivity of CCGC PDs with 0% and 20% Gd^3+^ alloying [75]; (**d**) schematic of the high-speed photodetector device based on Cs_4_CuSb_2_Cl_12_ NCs [76]; (**e**) ultrafast photocurrent peak density and corresponding dark current density as a function of bias voltage, measured from the same device [76]; (**f**) typical ultrafast photocurrent responses under various bias voltages. The insets show the dependence of photocurrent peak on bias voltage [76]. The figures were adapted from the cited references.

Among the family of all-inorganic lead-free perovskite variant NCs, lanthanide-based perovskite variant NCs have attracted great attention for UV detection due to their unique d-f/f-f electronic transitions. For instance, lead-free RE-based Cs_3_CeCl_6_ NCs were introduced by Min et al. as promising materials for UV detection owing to their unique photoelectric properties (Figure 8a) [75]. The incorporation of Gd^3+^ further increased the PLQY from 57% to 96%, along with enhanced phase and chemical stability. Attributed to the parity-allowed 4f-5d transition of Ce^3+^ ions, the Cs_3_Ce_1-x_Gd_x_Cl_6_ NCs exhibited a narrow absorption peak at 334 nm (UV-A region). Moreover, when fabricated into UVPDs (Figure 8b), the device with 20% Gd^3+^ alloying achieved excellent optimal performance with a responsivity of 0.195 A∙W^−1^ and a specific detectivity of 7.938 × 10^11^ Jones at 310 nm under −0.1 V bias (Figure 8c). DFT calculations further revealed that Gd^3+^ preferentially occupied the thermodynamically stable 4e Wyckoff site, suppressing concentration quenching and reducing exciton binding energy (from 289 to 237 meV), which facilitated the charge separation and extraction.

Layered NCs with direct band gaps are ideal for high-speed photodetection due to their ultrafast charge carrier dynamics. In 2020, Cai et al. reported the first colloidal synthesis of Cs_4_CuSb_2_Cl_12_ NCs via a hot-injection method with decoupled cation and anion precursors [76]. By tuning the Cu^2+^/Ag^+^ ratio in Cs_4_Cu_x_Ag_2−2x_Sb_2_Cl_12_ (0 ≤ x ≤ 1) NCs, a crystal structure transformation from cubic (x = 0, indirect band gap) to monoclinic layered (x = 1, direct band gap) was achieved, accompanied by a band gap narrowing from ~3.05 to 1.79 eV. Taking advantage of both the layered electronic structure and colloidal properties, the Cu-based NCs are applied in photodetectors (Figure 8d), demonstrating ultra-fast response (~150 ps) and narrow linewidth (~10 GHz). The direct band gap nature combined the excellent charge transport characteristics, these NCs exhibiting promising potential for high-frequency optical communication applications (Figure 8e,f).

Gas sensors based on lead-free perovskite NCs have gained attention for room-temperature detection of harmful pollutants, owing to their high surface-to-volume ratio, tunable surface chemistry, and sensitive optical/electrical responses. For instance, Casanova-Chafer et al. reported the first gas sensors using lead-free perovskite NCs (Cs_3_Cu_2_Br_5_ and Cs_2_AgBiBr_6_) supported on graphene, where graphene acted as a transducing element to enhance carrier transport [77]. The direct bandgap Cs_3_Cu_2_Br_5_ NCs exhibited superior sensing performance compared to the indirect bandgap Cs_2_AgBiBr_6_ NCs for detecting NO_2_ (ppb level), H_2_, NH_3_, and H_2_S. Specifically, Cs_3_Cu_2_Br_5_@graphene achieved a NO_2_ detection limit (LOD) of 8.5 ppb, a H_2_S LOD of 13.6 ppm, and a NH_3_ sensitivity of 21.39% ppm^−1^, which are below the threshold limit values (TLVs) for ambient monitoring. The sensing mechanism was correlated to the band gap type (direct band gap favoring fast charge transfer), exciton binding energy (quantum confinement enhancing exciton-gas molecule interaction), and defect tolerance (surface defects acting as electronic traps for gas adsorption). Notably, graphene support improved the stability of perovskite NCs against moisture and enhanced charge transport, enabling room-temperature operation with low power consumption.

In addition to gas sensing, the tunable optical properties of lead-free perovskite variant NCs make them also suitable for ratiometric optical thermometer applications. [78,79,80] Li et al. developed an ultrasensitive fluorescence thermometer based on Te^4+^-doped 0D scandium-halide perovskite Cs_2_ScCl_5_·H_2_O [81]. The unique electronic structural configuration enables bright STE emission with broadband orange emission. Notably, the STEs of Cs_2_ScCl_5_·H_2_O exhibit a sensitive temperature response. As temperature increases, the soft lattice undergoes asymmetric expansion, leading to enhanced defect generation, intensified exciton–phonon coupling, and a low thermal activation energy (102.11 meV). These properties collectively trigger rapid STE de-trapping, resulting in a several-order-of-magnitude change in PL lifetime over the temperature range of 300–370 K (670.2 ns to 3 ns). By optimizing the Te^4+^ doping concentration, the thermometer achieves a record-high thermal sensitivity of 27.36% K^−1^ at 325 K, which is one of the best lifetime-based fluorescent thermometers reported before. Practically, the thermometer enables remote temperature monitoring of internal electronic components (e.g., power management circuits and main control chips in USB flash drives) by penetrating non-transparent plastic shells.

### 3.3. Information Encoding

All-inorganic lead-free perovskite variant NCs have emerged as promising candidates for advanced information encoding applications, benefiting from their excellent optical tunability, robust stability, and diverse stimulus-responsive behaviors [82,83,84]. Stimulus-responsive luminescence switching, particularly water-triggered hydrochromism, is a core strategy for dynamic information encoding in lead-free perovskite variants.

**Figure 9:**
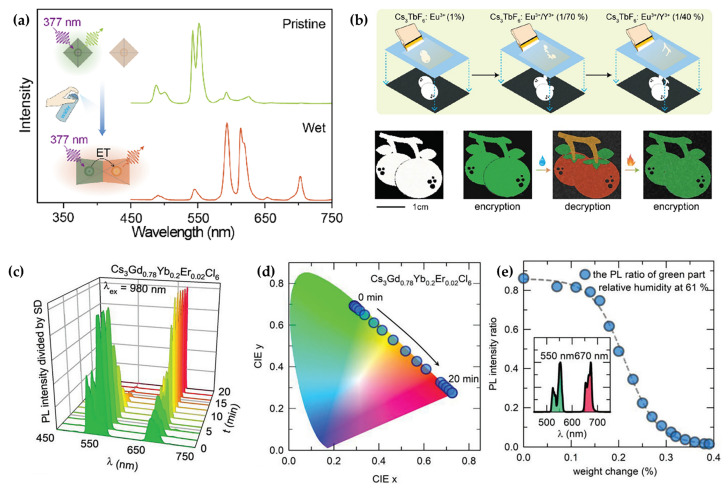
(**a**) Emission spectra of Cs_3_TbF_6_:Eu^3+^ before and after water treatment [85]; (**b**) schematic illustration of the multicolor pattern fabrication procedure via sequential screen printing [85]; (**c**) hydrochromic UCL of CGC:Yb^3+^,Er^3+^ under exposure to atmospheric moisture over time [40]; (**d**) shift in CIE chromaticity coordinates of the UCL as a function of exposure time to atmospheric moisture [40]; (**e**) variation in the ratio of green to red UCL intensity [40]. The figures were adapted from the cited references.

In 2022, Chen et al. reported lanthanide-doped Cs_3_TbF_6_:Eu^3+^ with remarkable potential for information encryption and anti-counterfeiting applications [85]. This class of rare-earth halides exhibits an ultra-fast response, undergoing water-triggered luminescence color switching within only 20 ms (Figure 9a), a rate significantly faster than that of known inorganic crystalline materials. Furthermore, by tuning the Eu^3+^ doping concentration and introducing Y^3+^ co-doping, the emission color can be finely adjusted across a wide spectral range, yielding multicolor output from green to orange and substantially enhancing the capacity and flexibility of information encoding. The material also shows excellent cyclic stability and environmental robustness, maintaining consistent luminescence performance after repeated wetting–drying cycles, under elevated temperatures, and during continuous UV irradiation, making it suitable for repeated use and long-term storage in practical settings. Additionally, the system supports a multi-stimuli responsive encryption mechanism. The output information can be controlled not only by water stimulation but also by selecting different excitation wavelengths (e.g., 377 nm and 393 nm), establishing an “excitation-stimulus” dual-factor encryption platform that significantly improves information security. The material can be further processed via screen printing and related techniques to produce multicolor patterns or quick response codes that display completely different optical information under dry and wet states, offering promising utility in high-level anti-counterfeiting labels and multi-level encrypted storage media (Figure 9b).

Another representative system is the 0D Cs_3_GdCl_6_:Yb^3+^,Er^3+^ metal halide, which integrates hydrochromism with photon upconversion (UCL) under 980 nm near-infrared (NIR) excitation [40]. This material exhibits a reversible UCL color change from green to red upon moisture absorption, attributed to non-radiative energy transfer from Er^3+^’s green-emitting levels to red-emitting levels mediated by water molecules (Figure 9c–e). The hydrochromic UCL property enables quantitative water detection in organic solvents (e.g., tetrahydrofuran) and flexible information encryption via laser handwriting. Laser-induced local water evaporation reveals hidden ciphertexts under NIR excitation, and the texts automatically disappear upon rehydration by atmospheric moisture. The excellent repeatability and non-contact excitation advantage make Gd-based NCs suitable for secure military and industrial information storage.

Excitation-wavelength-dependent multicolor emission provides an additional dimension for high-security information encoding, as the encrypted information can only be decoded under specific excitation conditions, effectively preventing counterfeiting and unauthorized access. Li et al. synthesized The Cs_2_NaRECl_6_ (RE = Tb, Eu) NCs via hot injection [86]. This series of materials demonstrates a unique dual-mode luminescence mechanism, offering considerable promise for information encryption applications. Cs_2_NaRECl_6_ (RE = Tb, Eu) exhibits both intrinsic broadband emission of the host matrix and characteristic narrow band emissions from the incorporated rare earth ions (Tb^3+^, Eu^3+^). By simply adjusting the excitation wavelength, the intensity ratio between these two emission components can be dynamically modulated, enabling tunable output colors ranging from red and green to blue. For instance, Cs_2_NaTbCl_6_ emits green light under 280 nm excitation but switches to blue emission when excited at 370 nm. Similarly, Cs_2_NaEuCl_6_ shows red emission under 300 nm excitation and blue emission under 370 nm excitation. This capability of generating multiple colors from a single material significantly simplifies the design and fabrication of anti-counterfeiting patterns while enhancing the concealment and decryption complexity. By combining the NCs with different rare earth compositions (e.g., Cs_2_NaEuCl_6_, Cs_2_NaTb_0.5_Eu_0.5_Cl_6_, and Cs_2_NaTbCl_6_), leaf-shaped patterns were fabricated that exhibit distinct color transitions from red/green to pink/blue, when switched between 302 nm and 365 nm excitation. Furthermore, a QR code prepared using Cs_2_NaTbCl_6_ NCs remains invisible under ambient light but becomes clearly readable under UV excitation and can be successfully scanned and decoded by a smartphone, realizing an integrated “light-controlled information storage and retrieval” anti-counterfeiting.

These applications highlight not only the flexibility of the material in visual anti-counterfeiting but also its innovative potential in optical information encryption. Compared with conventional static anti-counterfeiting materials, this class of NCs offers advantages such as fast response, high color purity, good photostability, and a relatively simple preparation process. Moreover, they avoid the toxicity issues associated with lead-based materials, rendering them more environmentally benign and safer for practical use.

Ultrafast fiber lasers have emerged as versatile platforms for both nanoscale optical encryption and high-speed, quantum-resistant physical-layer secure communication. Lead-free perovskite variant NCs have recently attracted attention as robust saturable absorbers (SAs) in this context [87]. In pioneering work, Cs_2_AgBiBr_6_ NCs enabled mode-locked Er-doped fiber lasers with ultrashort pulses (452 fs), low saturation intensity (0.75 MW/cm^2^), and a starting threshold as low as 50 mW. Beyond these exceptional nonlinear optical properties, the material demonstrates outstanding long-term stability, sustaining efficient mode-locking performance over ten months. These results highlight Cs_2_AgBiBr_6_ and related lead-free perovskites as promising candidates for next-generation ultrafast photonic devices that combine high performance with environmental friendliness and operational durability.

### 3.4. X-Ray Imaging

All scintillators serve as pivotal components in fields such as medical imaging and industrial non-destructive testing [88,89]. However, the conventional commercial scintillators (CsI:TI, LYSO:Ce) are hindered by their inherent mechanical rigidity, which complicates their processing into flexible films suitable for imaging curved or irregular surfaces. Additionally, these materials often suffer from limited light yield and a hygroscopic nature that leads to deliquescence. In contrast, lead-free halide NCs, which can be synthesized via low-temperature solution processes, exhibit high radioluminescence efficiency and can be readily fabricated into flexible films, presenting a promising alternative for next generation X-ray detection and imaging technologies [90].

In 2022, Li et al. reported for the first time the in situ precipitation of lead-free cesium manganese halide NCs (CsMnX_3_, X = Cl, Br, I) within a glass matrix and systematically evaluated their potential for X-ray scintillation imaging [60]. This series of Mn-based halides exhibits broad emissions with large Stokes shifts and negligible self-absorption. By adjusting the halide composition (Cl, Br, and I), the coordination environment of the Mn^2+^ ions can be precisely tuned from octahedral to tetrahedral, enabling wide-range luminescence color modulation from red (~640 nm) to green, which offers flexibility for matching different photodetectors. In terms of X-ray scintillation performance, the material demonstrates outstanding characteristics. The CsMnCl_3_ NC-embedded glass achieves an estimated light yield as high as 13,400 photons/MeV, significantly surpassing that of conventional CsPbBr_3_ glass composites and reaching a level comparable to that of commercial BGO single crystals. Its radioluminescence intensity exhibits a linear response over a broad dose-rate range (0.5–162.4 mGy_air_∙s^−1^), with a minimum detectable dose rate of 470 μGy_air_∙s^−1^. More importantly, scintillator screens fabricated from this material achieved a spatial resolution of 4.0 lp∙mm^−1^ in X-ray imaging and successfully resolved fine internal circuit structures of a chip, as well as a delicate spring with a diameter of only 180 μm inside a capsule. These results underscore its practical potential for high-resolution, low-dose X-ray imaging applications.

Moreover, emission wavelength tunability was achieved through halide alloying and cation doping (e.g., with Sb^3+^ or Mn^2+^), expanding the material platform for multicolor X-ray imaging and light-emitting devices.

**Figure 10:**
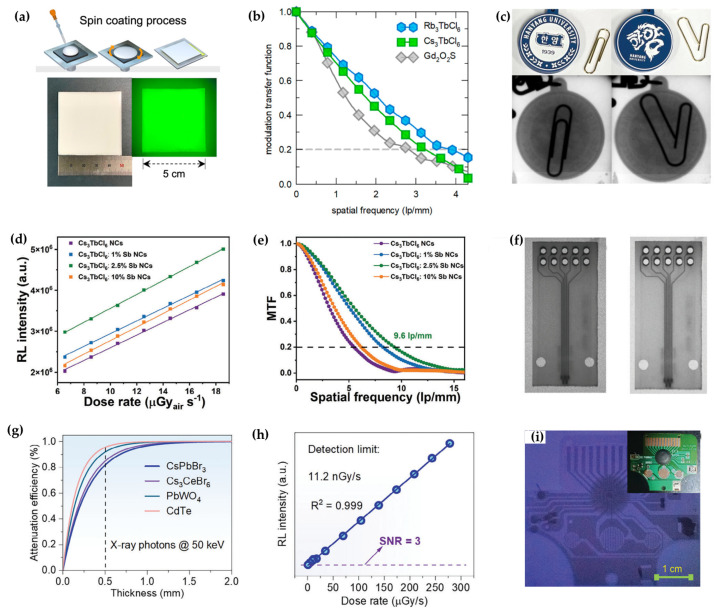
(**a**) Fabrication method of CTC and RTC scintillator films [91]; (**b**) spatial resolution of CTC, RTC, and GOS scintillator films [91]; (**c**) photographs of normal (left) and bent (right) clips and a key ring [91]; (**d**) dose rate dependence of the RL intensity of undoped and Sb-doped Cs_3_TbCl_6_ NCs [38]; (**e**) MTF of X-ray images from undoped and Sb-doped Cs_3_TbCl_6_ NCs [38]; (**f**) images of the target chip under X-ray imaging using a Cs_3_TbCl_6_:Sb nanoscintillator [38]; (**g**) attenuation efficiencies of CsPbBr_3_, PWO, CdTe, and Cs_3_CeBr_6_ [43]; (**h**) RL intensity of Cs_3_CeBr_6_ NCs [43]; (**i**) X-ray images of an electronic chip [43]. The figures were adapted from the cited references.

RE-based all-inorganic perovskite variants stand out for their narrow-band emission and high thermal stability, addressing the need for high-contrast X-ray imaging. In 2023, Han et al. developed a series of Tb-based 0D NCs Cs_3_TbCl_6_ and Rb_3_TbCl_6_ (Figure 10a) [91]. The sharp green emission from Tb^3+^ 4f–4f transitions not only enhances imaging contrast but also matches well with the spectral sensitivity of commercial photodetectors. Scintillator films based on Cs_3_TbCl_6_ and Rb_3_TbCl_6_ achieved spatial resolutions of 3.3 and 3.9 lp∙mm^−1^, respectively, surpassing that of the commercial Gd_2_O_2_S:Tb screen (Figure 10b). In static X-ray imaging, these films clearly resolved fine structures of shielded objects, demonstrating high contrast and detail-discrimination capability, which confirms their potential for both static and dynamic imaging applications (Figure 10c). Inspired by this work, our group synthesized Cs_3_TbCl_6_:Sb NCs via a modified hot-injection method [38]. The introduction of Sb^3+^ ions successfully created a thermally enhanced energy-transfer pathway, where [SbCl_6_]^3−^ octahedra induce self-trapped states as intermediate energy-transfer platforms. This strategy overcomes the limitation of inefficient energy-level matching in conventional rare-earth-doped systems, boosting the photoluminescence quantum yield to 48.1%. Furthermore, Sb doping markedly improved the X-ray scintillation properties, reducing the detection limit to 212 nGy_air_∙s^−1^ (Figure 10d), increasing the light yield to 20,300 photons/MeV, and achieving a spatial resolution of 9.6 lp∙mm^−1^ (Figure 10e). This work demonstrates that ns^2^-ion doping in rare-earth halide NCs can establish novel energy-transfer channels, enabling the integration of narrow-band, high-brightness luminescence with high-resolution scintillation (Figure 10f).

While rare-earth-based lead-free NC scintillators have achieved significant advances in X-ray imaging, the ms-scale long luminescence lifetime of their forbidden f-f transitions has limited their uses in fast, dynamic imaging. In 2025, Yang et al. reported Cs_3_CeBr_6_ NCs that simultaneously combine large-area processability, rapid response, and excellent scintillation performance (Figure 10g) [43]. The allowed 5d-4f transition of Ce^3+^ enables fast radiative recombination, yielding a decay lifetime of only ~29 ns, which is much faster than that of conventional lanthanide-based scintillators. This fast decay not only effectively eliminates “ghosting” artifacts in imaging but also makes the material suitable for real-time dynamic X-ray imaging. Furthermore, by introducing tri-n-octylphosphine (TOP) as a ligand, the surface passivation and colloidal stability of the nanocrystals were markedly improved, resulting in a PLQY close to 100% and providing a foundation for fabricating large-area flexible devices. Capitalizing on these favorable radioluminescence characteristics, X-ray imaging was demonstrated with a spatial resolution of 12.21 lp∙mm^−1^ and an ultra-low detection limit of 11.2 nGy∙s^−1^, both of which exceed conventional medical-imaging standards (Figure 10h). By embedding the NCs in a PDMS matrix, the researchers successfully fabricated flexible and stretchable scintillator films and demonstrated their potential for high-resolution static and dynamic X-ray imaging (Figure 10i). Scintillation properties of typical all-inorganic metal halide perovskite variant NCs were summarized in Table 2.

## 4. Conclusions and Outlook

All-inorganic lead-free perovskite variant NCs have emerged as a versatile and promising material platform for advanced photonic applications. By addressing the critical limitations of lead toxicity and environmental instability in traditional lead-based perovskites, these NCs have unlocked new opportunities across optoelectronics, biomedicine, radiation detection, and information technology. Nevertheless, the advancement of lead-free perovskite variants is met with both significant challenges and substantial opportunities.

**Synthesis refinement**

Despite significant advances in the research of lead-free perovskite nanocrystals in recent years, their synthesis remains largely confined to the hot-injection and ligand-assisted reprecipitation (LARP) methods. While hot injection offers precise control over nanocrystal morphology and crystallinity through regulation of reaction temperature and duration, it typically requires an inert atmosphere and elevated reaction temperatures. The LARP approach, on the other hand, still presents considerable room for improvement in terms of product uniformity, crystallinity, and defect control. Furthermore, both methods face challenges related to high cost and difficulties in large-scale production, which pose bottlenecks to their commercial adoption. Developing more efficient synthetic strategies—such as ultrasound-assisted synthesis—that can ensure high nanocrystal performance while enabling green, low-cost, and scalable fabrication represents a critical challenge and a key direction for future research.

**Luminescence mechanism exploration and efficiency optimization**

Currently reported luminescence mechanisms in lead-free perovskite nanocrystals primarily include free-exciton emission, self-trapped exciton emission, defect-related emission, and transitions from specific energy levels of dopant ions. Within a single host lattice, multiple emissive centers can coexist, yet the interactions among these centers require deeper mechanistic understanding. Employing advanced spectroscopic techniques, such as femtosecond transient absorption spectroscopy and synchrotron-radiation-based spectroscopy to characterize these ultrafast processes and clarify the energy-transfer pathways in lead-free nanocrystals is highly valuable. Such insights will not only further improve their photophysical performance but also broaden their applications in optoelectronic devices.

**Stability improvement**

While lead-free all-inorganic perovskite variant nanocrystals circumvent the toxicity of lead and the instability of organic components, their exceptionally high surface-to-volume ratio predisposes them to a high density of defect states. These defects not only act as non-radiative recombination centers that diminish luminescence efficiency but also serve as channels for moisture/oxygen ingress and ion migration, thereby accelerating material degradation. Furthermore, nanocrystals are inherently more vulnerable than their bulk or thin-film counterparts to environmental factors such as light, heat, moisture, and oxygen, which exacerbate lattice hydrolysis and decomposition. Therefore, developing more effective strategies to enhance their stability is imperative. For instance, approaches include compositional engineering, such as B-site alloying and heterovalent doping at A- or B-sites, which can modulate the crystal field, increase the formation energy, suppress detrimental phase transitions, and simultaneously passivate defects. Alternatively, constructing core–shell architectures can physically isolate the active core from ambient water and oxygen, while also suppressing ion migration and surface degradation.

**Application Expansion**

In recent years, breakthroughs in lead-free perovskite NCs have rapidly expanded their application scope far beyond the initial focus on light emission, driving their adoption in diverse and high-performance advanced photonics and interdisciplinary fields. These emerging applications span pixelated electroluminescence, high-performance photodetection and imaging, nanolasers, quantum light sources, and photocatalysis. Looking forward, by further exploring their multifunctional properties and integrating them with established systems such as organic materials and silicon photonics, it is promising to construct a new generation of high-performance, integrated photonic platforms.

## Figures and Tables

**Table 1:** Optical properties of lead-free perovskite variant NCs.

Perovskite NCs	λ_em_ [nm]	Stokes Shift [nm]	PLQY [%]	Emission Mechanism	Reference
Cs_2_AgInCl_6_:Bi	565	194	2.3	STEs	[54]
Cs_2_AgInCl_6_:Na/Bi	575	207	56.5	STEs	[55]
Cs_2_AgInCl_6_:Eu	612	401	N/A	STEs to Eu^3+^: ^5^D_0_ → ^7^F_J_ transitions	[56]
Cs_2_AgInCl_6_:Al	600	290	8.4	STEs	[57]
Cs_2_NaInCl_6_:Rb,Sb	528	208	38.6	STEs	[58]
Cs_2_KInCl_6_:Sb	515	195	95	(1) Direct E_g_ (2) Energy transfer from a nonradiative singlet state	[59]
CsMnBr_3_	643	99	54	^4^T_1_ → ^6^A_1_ transition	[24]
CsMnCl_3_	644	121	41.8	STE states and ^6^A_1_ state of Mn^2+^ ions	[60]
Cs_3_MnBr_5_	520	60	48	^4^T_1_ → ^6^A_1_ transition	[23]
Cs_4_MnSb_2_Cl_12_	640	270	0.5	^4^T_1_ → ^6^A_1_ transition	[25]
Cs_4_MnBi_2_Cl_12_	619	249	0.5	[BiCl_6_]^3−^ to Mn^2+^ ions and the consequent d-d transition	[61]
Cs_2_ZrBr_6_	513	225	40	STEs	[62]
Cs_2_ZrCl_6_	446	201	60.37	STEs	[63]
Cs_2_HfCl_6_	378	138	1.39	STEs	[64]
Cs_2_TiBr_6_	580	100	N/A	STEs	[65]
Cs_2_SnI_6_	790	N/A	1.36	STEs	[66]
Cs_2_SnCl_6_	444	N/A	0.4	STEs	[66]
Cs_2_SnBr_6_	600	255	31	STEs	[67]
Cs_3_Cu_2_Cl_5_	510	200	≈100	STEs	[68]
Cs_3_Cu_2_Br_5_	454	177	18.3	STEs	[27]
Cs_3_Cu_2_I_5_	441	144	67	STEs	[27]
CsCu_2_I_3_	553	229	5	STEs	[27]
Cs_3_TbCl_6_:Sb	548	225	48.14	^5^D_4_ → ^7^F_6_ transition	[38]
Cs_2_NaTbCl_6_:Ce	548	205	21	^5^D_4_ → ^7^F_J_ transitions	[69]
CsEuCl_3_	435	70	5.7	Band edge	[33]
Cs_3_EuCl_6_ (EHC)	617	252	92.4	^5^D_0_ → ^7^F_2_ transition	[35]
Cs_3_CeBr_6_	390, 425	40, 75	67.3	^5^D_1_ → ^2^F_5/2_, ^5^D_1_ → ^2^F_7/2_ transition	[43]
Cs_3_YbCl_6_	429	55	67.3	STEs	[43]

**Table 2:** Scintillation properties of typical all-inorganic metal halide perovskite variant NCs.

Material	Emission Peak [nm]	Decay Time [μs]	Light Yield [Photons∙MeV^−1^]	Direction Limit [n∙Gy∙s^−1^]	Spatial Resolution [lp∙mm^−1^]	Reference
Cs_3_Cu_2_I_5_	445	1.92	39,251	815	100	[92]
Cs_3_Cu_2_Cl_5_	520	95.79	34,000	81.7	9.6	[93]
Rb_2_CuCl_3_	400	9.9	16,600	88.5	N/A	[94]
Rb_2_CuBr_3_	387	46.7	91,056	121.5	N/A	[94]
Cs_5_Cu_3_Cl_6_I_2_	446	39	66,000	11	27	[95]
Cs_3_TbCl_6_	548	5400	13,200	1817	5.5	[38]
Cs_3_TbCl_6_:Sb	548	7600	20,300	212	9.6	[38]
Cs_2_NaTbCl_6_	548	5000	14,800	700	4.4	[34]
Rb_3_TbCl_6_	548	4380	88,800	115.38	3.9	[91]
Cs_2_NaEuCl_6_	593	14,330	1250	N/A	N/A	[96]
Cs_2_ScCl_5_·H_2_O:Tb	548	1515.9	N/A	N/A	9.5	[97]
Cs_2_NaBiCl_6_:Mn	586	1220	28,350	45.2	14.76	[98]
CsCdCl_3_:Mn	605	7920	N/A	N/A	12.2	[99]
CsMnCl_3_	630	376	13,400	470	4.0	[100]
Cs_2_ZrCl_6_	440	83.55	49,400	65	18	[101]
Cs_2_HfCl_6_	447	N/A	21,700	55	11.2	[102]
Cs_2_AgIn_0.9_Bi_0.1_Cl_6_	550	N/A	32,000	87	12	[103]
Cs_3_CeBr_6_	425	0.029	N/A	11.2	12.21	[43]

## Data Availability

No new data were generated or analyzed in this study. All data discussed in this review are available in the cited literature.

## Abbreviations

The following abbreviations are used in this manuscript:

0D	Zero-dimensional
1D	One-dimensional
3D	Three-dimensional
BGO	Bismuth germanate
CIE	Commission Internationale de I’Éclairage
DFT	Density functional theory
EHC	Ethylhexyl substituted coumarin
EL	Electroluminescence
EQE	External quantum efficiency
FWHM	Full width at half maximum
Gd	Gadolinium
LARP	Ligand-assisted reprecipitation
LED	Light-emitting diode
LF-PNCs	Lead-free perovskite nanocrystals
LHP	Lead halide perovskite
Ln	Lanthanide
LOD	Limit of detection
LYSO:Ce	Lutetium-yttrium oxyorthosilicate doped with cerium
MTF	Modulation transfer function
NC	Nanocrystal
NIR	Near-infrared
ns^2^	ns^2^-type ions (e.g., Sb^3+^, Bi^3+^, Te^4+^)
NTSC	National Television System Committee
PCE	Power conversion efficiency
PD	Photodetector
PDMS	Polydimethylsiloxane
PeLED	Perovskite light-emitting diode
PL	Photoluminescence
PLE	Photoluminescence excitation
PLQY	Photoluminescence quantum yield
PMA	Phosphomolybdic acid
ppb	Parts per billion
ppm	Parts per million
ps	Picosecond
PWO	Lead tungstate
QR code	Quick response code
RE	Rare earth
Rec. 2020	Recommendation ITU-R BT.2020
RL	Radioluminescence
STE	Self-trapped exciton
TLV	Threshold limit value
TMSX	Trimethylsilyl halide
TOP	Tri-n-octylphosphine
TR-PL	Time-resolved photoluminescence
TRPL	Time-resolved photoluminescence
UCL	Upconversion luminescence
USB	Universal serial bus
UV	Ultraviolet
UVPD	Ultraviolet photodetector
X	Halogen (Cl, Br, I)
μGy	Microgray
